# Multi-Sensor Fusion for Enhanced Contextual Awareness of Everyday Activities with Ubiquitous Devices

**DOI:** 10.3390/s140305687

**Published:** 2014-03-21

**Authors:** John J. Guiry, Pepijn van de Ven, John Nelson

**Affiliations:** Department of Electronic & Computer Engineering, University of Limerick, Limerick, Ireland; E-Mails: pepijn.vandeven@ul.ie (P.V.); john.nelson@ul.ie (J.N.)

**Keywords:** sensor fusion, ubiquitous activity monitoring, smart devices, smartphone, smartwatch, geospatial awareness, activities of daily living

## Abstract

In this paper, the authors investigate the role that smart devices, including smartphones and smartwatches, can play in identifying activities of daily living. A feasibility study involving N = 10 participants was carried out to evaluate the devices' ability to differentiate between nine everyday activities. The activities examined include walking, running, cycling, standing, sitting, elevator ascents, elevator descents, stair ascents and stair descents. The authors also evaluated the ability of these devices to differentiate indoors from outdoors, with the aim of enhancing contextual awareness. Data from this study was used to train and test five well known machine learning algorithms: C4.5, CART, Naïve Bayes, Multi-Layer Perceptrons and finally Support Vector Machines. Both single and multi-sensor approaches were examined to better understand the role each sensor in the device can play in unobtrusive activity recognition. The authors found overall results to be promising, with some models correctly classifying up to 100% of all instances.

## 1. Introduction

Pervasive, ubiquitous computing is coming ever closer, and the implications for user driven preventative healthcare are immense. Modern smartphones and related devices now contain more sensors than ever before. Microelectromechanical Systems (MEMS) have made many leaps in recent years, and it is now common to find sensors including accelerometers, magnetometers and gyroscopes in a variety of smart devices. The addition of these sensors into everyday devices has paved the way towards enhanced contextual awareness and ubiquitous monitoring for healthcare applications.

The pervasive nature of these devices becomes particularly apparent when reviewing the rate of global sales. Total smartphone sales alone are estimated to have surpassed 837 million units in 2013 [1]. Furthermore, a worldwide growth rate of 26% has been predicted for both tablets and smartphones between 2012 and 2016. These devices are inherently portable, and consequently remain in close proximity to the user over long periods of time. Kearney [2] estimated that by the year 2017, OECD countries will save $400 billion from yearly healthcare costs, due to adopted mobile health solutions.

Furthermore, the reach of such devices is not constrained to developed economies alone. The International Telecommunication Union [3] estimates that there are 6.8 billion mobile subscriptions globally, comprised of subscription rates of 128.2% and 89.4% for those in developed and developing nations respectively. Mobile devices provide an effective healthcare solution for remote monitoring of those most at risk. Thus there is a compelling case to evaluate the effectiveness of such devices in healthcare related applications.

Several uses exist for such a device, including fall detection [4–7] and fall prevention [8,9]. One particularly relevant application involves monitoring everyday physical activities and sedentary behaviour. A recent *Lancet* publication [10] estimates that physical inactivity alone causes 9% of all premature deaths worldwide. This figure represents over 5.8 million deaths in 2008. Furthermore, eradicating physical inactivity would increase life expectancy of the world's population by an average of 0.68 years.

This sentiment is echoed in publications by the U.S. Department of Health and Human Services, which found a strong correlation between increased physical activity and a lower risk of heart disease, stroke, high blood pressure, type II diabetes and even particular forms of cancer. Research conducted by Heidenreich *et al.* [11] and Dall *et al.* [12] documents the financial burden caused by such diseases. Heidenreich *et al.* found the total cost in 2010 of coronary heart disease among Americans to be $108.9 billion, while Dall *et al.* estimated the 2007 cost of Americans suffering type II diabetes to be in excess of $159 billion. Furthermore, the prevalence of cardiovascular disease and stroke is predicted to increase by an average of 20.75% among the American populous by 2030. A similar report by Leal *et al.* [13] places the 2003 total cost of coronary heart disease in the EU area at €44.7 billion ($56.5 billion, at 2003 rates [14]), which includes €294 million ($371 million, at 2003 rates [14]) for Ireland.

A number of studies have already examined the potential role single sensors can play in activity recognition, including the accelerometer [15–18] and GPS [19]. More recently, the use of multiple sensors has come to the fore, e.g., [20,21]. In this scenario, a smart device, such as a mobile phone can either act as gateway for one or more dedicated devices located in a Personal Area Network (PAN), e.g., [22], or the sensors built into the smartphone can be used, e.g., [23].

In this paper, we examine the potential role smartphones and smartwatches can play in the inference of everyday human ambulation using both single and fused sensor approaches. We also investigate the potential of using both GPS and light sensors to better infer when patients have transitioned from indoors to outdoors or *vice versa*. To this end, the focus is set firmly on the built in sensors available on these devices. Section 2 details some related work in the field, while Sections 3 and 4 describe the sensor setup and signal processing undertaken as part of this research experiment. Section 5 details the features computed from the raw sensors, and used for subsequent training of machine learning models. Section 6 provides a description of how the study was carried out, and details of the cohort are also provided. Section 7 presents a discussion of results attained from the study data. Finally, Section 8 outlines a conclusion and describes areas where work still remains to be done.

## 2. Related Work

A number of papers have attempted to gather and infer physical activities using dedicated sensors, often strapped to the user using belts or tape, e.g., [24–30]. Recently, the viability of smartphones to perform the same role, yet in a less obtrusive sense, has become more apparent.

Kwapisz *et al.* [31] use an Android-based cell phone accelerometer to collect data from 29 participants. Data was collected at 20 Hz, and used to train three machine learning models: J48, Logistic Regression and a Multilayer Perceptron. Activities tested included walking, jogging, going up and down stairs, sitting and standing. Moving up and down stairs proved to be most difficult to detect, with best accuracies of 55% and 61%, respectively. However, the authors only examined the use of a cell phone accelerometer. No data was collected from any other sensor in the trial.

Maurer *et al.* [32] used a bi-axial accelerometer together with a light sensor on a dedicated eWatch sensing platform to record six activities: standing, sitting, running, walking, ascending and descending stairs. The authors achieved accuracies of up to 92%, though it is unclear if this was based on a balanced or unbalanced dataset. Devices were limited to 1 MB of flash memory.

Ganti *et al.* [21] recorded data from four sensors using a Nokia N95 device. These included the microphone, accelerometer, GPS and GSM (for additional location based information). The accelerometer sensor was sampled at 7 Hz, while the microphone was sampled at 8 kHz. Eight distinct activities were recorded, including aerobic, cooking, desk work, driving, eating, hygiene, meeting and watching television. Features chosen included estimates of energy expended, skewness of acceleration magnitude, and the cepstral coefficients computed from the microphone data. The authors chose to use a three state Hidden Markov Model (HMM) which gave average results of 66%.

## 3. Sensor Setup

**Figure 1.**
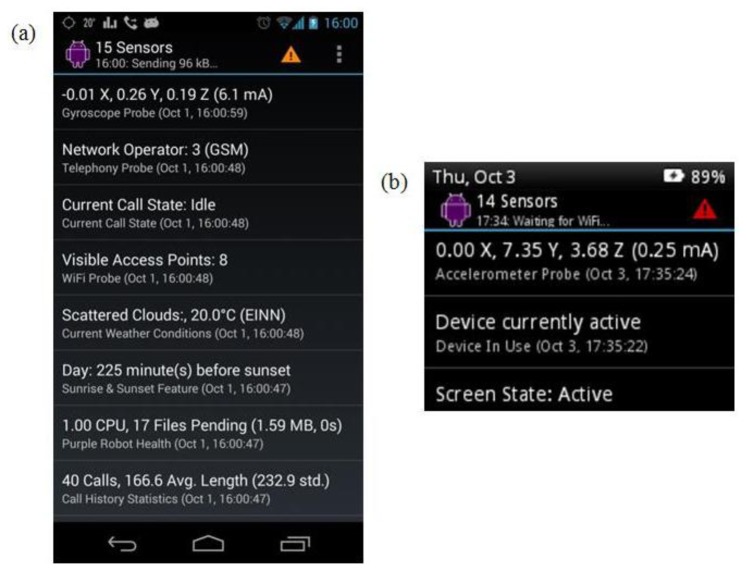
(**a**) Android application running on smartphone; and (**b**) smartwatch.

Both a Samsung Galaxy Nexus smartphone and the Motorola MotoActv smartwatch were used to gather data from all possible sensors. On the Nexus, data was obtained from the tri-axial accelerometer, tri-axial magnetometer, tri-axial gyroscope, GPS, light and pressure sensor. On the smartwatch, data was collected from the tri-axial accelerometer, in part due to the fact that this was the only activity-related sensor available on this device. The authors used Purple Robot to gather data on both devices, as depicted in Figure 1. This Android application, developed by the Centre for Behavioural Interventions at Northwestern University, gives researchers access to dozens of underlying device sensors. Known by the term “probes” in Purple Robot terminology, these represent both physical and virtual sensors. Such probes can include accelerometers, gyros, and message and call statistics. Purple Robot uses a store and forward mechanism, only uploading data to the Purple Robot warehouse server, when a suitable data connection becomes available. In our experiments, the Wi-Fi connection was used once data collection was complete to upload all sensor data pertaining to the study. Both devices were linked via a separate application running on the researcher's phone. This application, called the Syncatronic, was used to annotate activities in the moment, and keep sensor data from both devices in sync for post hoc analysis.

## 4. Signal Processing

**Figure 2.**
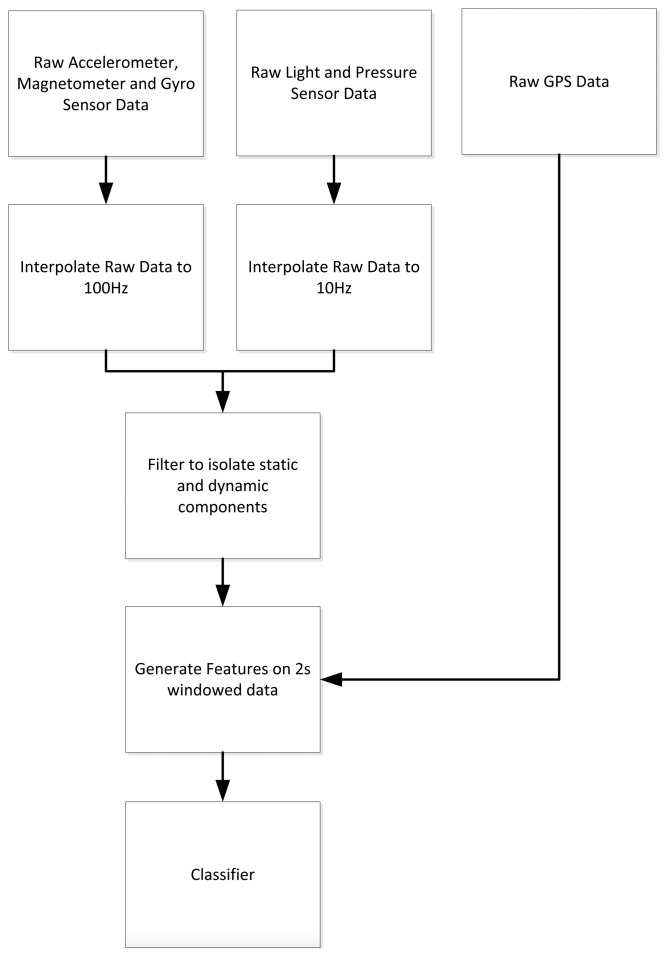
Signal processing flow diagram.

Initial data processing is undertaken to interpolate the raw accelerometer, magnetometer, and gyro signals to a rate deemed acceptable for subsequent filtering, as presented in Figure 2. On both devices, raw accelerometer data can be somewhat sporadic, approximating 90 Hz and 15 Hz on the phone and watch, respectively. Unlike some predecessors to the Nexus smartphone, the authors have no issues to report regarding sensors unexpectedly powering down, or an excessively low sampling rates. Due to the limited battery power of the smartwatch, the accelerometer was shut down during periods of little or no activity, *i.e.*, when the participant was typically sedentary. This prevented needless transmission of additional information to the server and had no negative effects on the study. In these circumstances, the last known good value from the accelerometer is held until the subsequent bout of movement. Both signals were linearly interpolated to a common, fixed sampling rate of 100 Hz for the purpose of subsequent analysis and feature generation. Similarly, the magnetometer, gyroscope and pressure sensor data sourced from the smartphone have raw sampling rates approximating 25 Hz, 27 Hz, and 5 Hz, respectively. Both magnetometer and gyroscope are interpolated to a fixed sampling rate of 100 Hz, while the pressure sensor is interpolated to a rate of 10 Hz. Data attained from the light sensor was interpolated to 10 Hz, while readings sourced from the GPS module were not interpolated.

With interpolation complete, the authors next focused on signal filtering. The applicability of such filtering is not particularly trivial. Both accelerometers were low- and bandpass-filtered for the purpose of isolating the dynamic components due to human movement, from the static components due to gravity. A low pass filter implementation was used, with a cut off of 0.6 Hz, for the static component, while cut offs of 0.6 to 7.5 Hz were used for the upper and lower boundaries of our band pass filter. Filtering is also applied to raw signals sourced from the gyroscope, magnetometer, and pressure sensor. Although gravity is not an issue for the magnetometer or gyroscope, it was decided to bandpass filter these between 0.6 and 7.5 Hz. These frequency cut offs were arbitrarily chosen to eliminate those components of the signal whose periodicity was less than or greater than typical human gait. Similarly, the pressure sensor accepts all frequencies above 0.1 Hz. For this sensor, a choice of 0.1 Hz is made to reduce the effects of expected daily pressure fluctuations caused by current atmospheric conditions.

**Figure 3.**
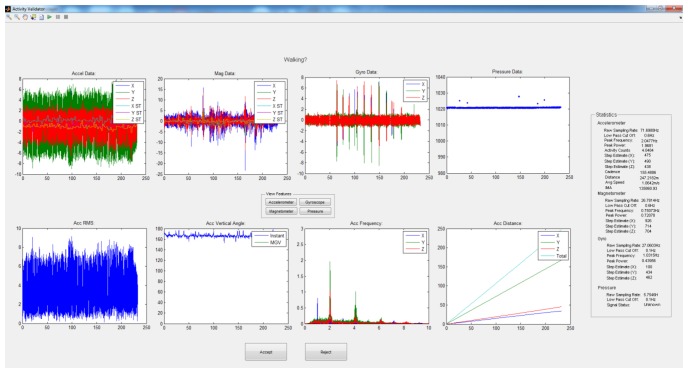
LightSabre application.

With preliminary signal processing complete, the signal is validated using a Matlab based application where each annotated activity can be either accepted or rejected, as depicted in Figure 3. This gives the researcher an opportunity to omit those segments which may have been mis-annotated, or otherwise unusable. In total, 354 segments were accepted from 486, combined from both the smartwatch and smartphone. The vast majority of rejected segments were omitted as they were “false starts”, *i.e.*, the researcher momentarily pressed the annotate button when preemptively waiting for the participant to begin that activity. To eliminate unnecessary lead time, the authors attempted to annotate activities as instantaneously as possible.

## 5. Feature Generation

Each segment in the array is windowed and features are generated from these windows. The authors used a window size of two seconds. For the phone, a comprehensive set of activity features can be found in Table 1, and include features sourced from the accelerometer, magnetometer, gyro and pressure sensors. Features generated included activity counts, device angle from the accelerometer and magnetometer, peak frequency and peak power for the magnetometer, gyro and accelerometer. Estimates of distance travelled, derived from the accelerometer, and altitude changes, derived from the pressure sensor, are also included.

A subset of the features documented in Table 1 was used for the smartwatch, specifically those pertaining to the accelerometer. These include activity counts, peak frequencies, peak power, estimates of the step count, the instantaneous angle, and the primary frequency where most of the power is concentrated.

Finally, the authors also generate features using the GPS and light sensors on the phone, to assist in detecting transitions from outdoors to indoors and vice versa. Features obtained from these sensors are documented in Tables 2 and 3. These include mean satellite count, mean satellite Signal to Noise Ratio (SNR), and mean lux before and after a transition.

## 6. Feasibility Study

**Figure 4.**
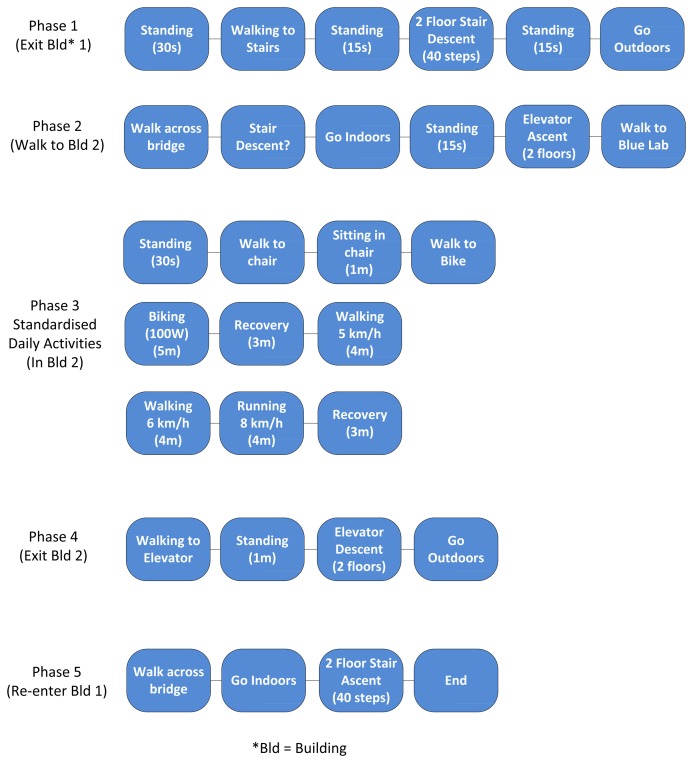
Protocol used in feasibility study.

The feasibility study was composed of a cohort of ten healthy participants (eight M, two F), with a mean age of 23 years. The protocol used in this study is depicted in Figure 4. As can be seen from this figure, activities recorded included sitting, standing, walking, running, cycling, stair descent, stair ascent, elevator descent and elevator ascent. Approximate times have been allocated to each activity.

Participants were each given a Galaxy Nexus smartphone, together with a Motoactv smartwatch. Participants were asked to place the smartphone in a pants pocket, while the smartwatch was placed on either wrist. The researcher never stipulated orientation of the smartphone or the pants pocket in which this should be placed. Similarly, the wrist on which the smartwatch was worn was left entirely at the discretion of participants. The researcher documented activities partaken using a separate smartphone running annotation and sensor synchronization software.

## 7. Discussion

Five de-facto machine learning algorithms were adopted to train and test from the generated features. These were C4.5, and CARTbased decision trees, Naïve Bayes, Multi-Layer Perceptrons and finally Support Vector Machines. Multiple datasets were created, including a dataset for both balanced and unbalanced phone and watch data. The authors use the term balanced here to refer to the case whereby each activity is assigned an equal number of instances. In the case of the work presented in this paper, each activity can contribute up to 11.1% of the overall true positive percentage presented in Tables 4 and 5, as there are a total of nine activities. Thus, a balanced dataset can be useful to highlight those activities which the classifier finds most difficult to infer, which may otherwise go unnoticed in the reported overall true positive rate of an unbalanced dataset. It also better reflects overall performance of the classifier to infer each activity. In the case of an unbalanced dataset, overall true positive rates may be skewed in favor of the activity which participants partook in most often, typically walking. While unbalanced datasets reflect the protocol and a real world scenario quite well, the authors still found balancing the dataset useful to give all activities an equal representation, and thus establish which activities were most difficult to infer for each of the models. It should be kept in mind that this is not reflective of human ambulation; humans do not spend equal amounts of time walking as climbing stairs for instance. Details of the results attained from these algorithms using the aforementioned datasets follows.

### 7.1. Smartphone

On the phone, the challenge was to differentiate between nine key activities. These were sitting, standing, walking, running, cycling, stair ascents, stair descents, elevator ascents and elevator descents. For activities pertaining to ascents and descents, a measurement of altitude calculated using data from the raw barometric sensor, was initially thought to be most relevant. However, due to the quality of the pressure sensor, this was not the case. This sensor accompanying all Galaxy Nexus phones supports a wide range from 300 to 1,100 mmHg. The authors noted from visualizing the pressure and altitude signals, that these did reflect the rising and falling nature of elevators and stairs quite well. However, there were also a significant number of false positives, even while participants stood still. This is likely caused by the fact that a change of 1 mmHg equates to a corresponding change of 8.4 m in altitude. The BMP 180 found in the Galaxy Nexus smartphone does not appear to be sensitive enough to detect pressure differentials experienced while partaking in these activities.

The data was analyzed from two perspectives: the first looked at the result attainable using the fused sensor data to infer all nine activities. Overall classification rate for these activities was as high as 94%, for all but the Naïve Bayes classifier, which attained a markedly lower result of 65%, as presented in Table 4.

Next, the authors investigated the role played by each of the other sensors on the smart phone. As one might expect, results attainable using just the accelerometry based features are almost identical to the fused results. In fact, the classification rate for the Naïve Bayes classifier increases significantly from 65% for the fused sensors, to 85% for the sole accelerometer. Of interest too are the overall classification rates achievable using either the magnetometer or the gyroscope. Results for these were as high as 89% and 79% respectively, demonstrating that these sensors can be used quite successfully when identifying activities in our unbalanced dataset. As one might expect, using the pressure sensor to solely identify activities was never greater than 47%.

With analysis on the unbalanced dataset complete, the authors decided to balance the dataset, to get a better understanding of which activities the classification algorithms struggled most to infer. Thus the unbalanced dataset was subsampled such that each activity was represented in this new balanced dataset with an equal number of instances. The best overall fused results dropped from 95% to 75%, a decline of some 20%. Similarly, best results attainable when using the magnetometer and gyroscope fell to 53%, and 43% respectively. Overall results of 20% attainable using the pressure sensor re-emphasize our earlier claim that this is too insensitive for activity recognition.

### 7.2. Smartwatch

Results from the smartwatch located on the wrist proved particularly fruitful. Overall activity recognition rates were 89% for all nine activities, when using the unbalanced dataset, as depicted in Table 5. Of particular use is the vertical angle feature, which could easily differentiate walking from running, and also help infer when stair climbing activities occurred. When the dataset is balanced, the C4.5 classifier comes out in top place, correctly inferring 56.89% of all activities, as presented in Table 5. This is a good result given that four of the nine activities were thought to rely heavily on an altitude sensor, which was not available on the watch. The smartwatch accelerometer was sensitive enough to differentiate when participants placed an arm on a support railing while climbing up or down stairs. Despite the fact the cohort examined were young, healthy participants, most reached out to the support railing.

### 7.3. Principal Component Analysis

Principal Component Analysis (PCA) is a technique used to reduce the dimensions of feature vectors. A principal component will follow the direction of the data with the largest variation, or power. The PCA algorithm is described fully in the literature, including [33] and involves the computation of the covariance matrix, eigenvalues and eigenvectors. PCA was applied to data from both watch and phone. From a total of 53 attributes, generated on the phone and defined earlier, analysis found that 29 of these features can be combined to cover 95% of the variance in the input values. Interestingly features generated from all three sensors (accelerometer, magnetometer, and gyroscope) all feature strongly in the top three principal components. Results attained when using this subset can be found in Table 6. Similarly, PCA was carried out on all 17 accelerometry features generated on the watch. Of these, 12 features were found to contain 95% of the attribute variance, and these were again provided to all five classifiers. Classification results attained when using this subset is presented in Table 7.

### 7.4. Outdoors vs. Indoors

The protocol was designed such that individuals partook in scripted activities in two separate buildings. The walk between buildings was outdoors and took between 5 and 10 min for all participants. During these tests, data was collected from the smartphones GPS and light sensors, to examine if these could be used to better differentiate between indoors and outdoors. This experiment was conducted during daylight hours. Results when using just GPS, just light, or the fused approach of using both are presented in Table 8. Depending on the classifier selected; results were as high as 95% and 88% when using the GPS and light sensors respectively. Using the Naïve Bayes classifier, the authors attained a 100% true positive classification rate when using data fused from both sensors.

## 8. Conclusions and Future Work

In this paper, the authors investigated the use of both smartphones and smartwatches as a means to infer physical activity, while enhancing geospatial awareness. Both smartphone and smartwatch provided valuable sensor data for the nine activities presented, and subsequent classification of features generated from this data attained results of up to 95% and 89% for smartphone and smartwatch respectively. The potential role of single versus multiple sensors was also established in this paper. While it is clear that the accelerometer contributes more than the others, it is also evident that sensors such as the gyroscope and magnetometer are capable of detecting a subset of activities quite well. The fusion of multiple sensors across a single device can prove beneficial in certain circumstances. This was reinforced when fusing both light and GPS sensors to successfully differentiate between indoor and outdoor activities using the Naïve Bayes classifier. Fully understanding when participants are indoors or outdoors can be useful to better comprehend a participant's physical and emotional wellbeing. Work is already underway to run calibration routines on the devices themselves. Such calibration routines can have positive implications on the models developed, and be tailored to the individual. Sensor calibration, on a per-user scale would allow a generalizable framework to be ported to other populations, such as the elderly or those with some gait abnormalities. The use of a pressure sensor to determine altitude differences may prove more beneficial once the quality of these sensors improve. Finally, there is some scope to investigate the use of smarter classifiers, potentially tiered which could choose between sensors, to optimize the balance between contextual knowledge and energy efficiency.

## Figures and Tables

**Table 1.** Features computed from smartphone data.

**Feature**	**Brief Description**	**Derived from** *
Activity Counts × 6	Activity counts are derived from the accelerometer and magnetometer, and indicate intensity. Activity Counts are output for each of the X, Y and Z axes.	A, M
RMS Counts × 2	Counts generated from the Root Mean Square of the accelerometer and magnetometer signals.	A, M
Mean Uncorrected Device Angle × 2	The mean angle, over a given time period. The vertical angle is taken to be the Y axis. This is derived from both accelerometer and magnetometer signals.	A, M
Mean Corrected Device Angle × 1	The corrected device angle is derived from the mean gravity vector of the accelerometer.	A
Coefficients of Variation × 6	The Coefficients of Variation derived from the accelerometer and magnetometer for X, Y and Z axes.	A, M
Max Power × 9	The maximum power derived from the accelerometer, magnetometer, and gyro signals. Three values are returned for each sensor, representing the X, Y and Z axes.	A, G, M
Peak Frequency × 9	The location in Hertz of the peak in the frequency spectrum for each of the X, Y and Z axes derived from the accelerometer, magnetometer, and gyro.	A, G, M
Peak Power × 3	The max value in the Max Power array, which will give an overall indication of intensity.	A, G, M
Primary Frequency × 3	The frequency which contains the most activity.	A, G, M
Step Count × 9	An estimate of the number of cyclical peaks in each axes.	A, G, M
Estimated Distance × 1	An estimate of the distance travelled in all 3 axes.	A
Altitude Difference × 1	The first order differential of altitude values (the current value less the prior value).	P
Mean Slope × 1	The mean slope of the altitude.	P

* A = Accelerometer, M = Magnetometer, G = Gyro, P = Pressure.

**Table 2.** Features computed from the smartphone's GPS module.

**Feature**	**Brief Description**
Mean Bearing	The average bearing while indoors or outdoors
Mean Speed	The average speed while indoors or outdoors
Mean Altitude	The average altitude while indoors or outdoors
Mean Satellite Count	The average number of visible satellites while indoors or outdoors
Mean Satellite SNR	The average satellite signal to noise ratio while indoors or outdoors

**Table 3.** Features computed from the smartwatches light sensor.

**Feature**	**Brief Description**
Raw Mean Lux	The mean light value attained from the raw signal
Low Pass Mean Lux	The mean light value attained from the low passed signal
Mean Differential Lux	The mean differential of the light signal

**Table 4.** Smartphone-based activity recognition.

**Balanced Dataset**					

	**Fused**	**Acc**	**Mag**	**Gyro**	**Pressure**
C4.5	70.97%	70.13%	45.55%	35.69%	14.16%
CART	68.61%	66.52%	51.25%	43.05%	19.44%
MLP	65.55%	61.38%	41.38%	36.80%	18.61%
SVM	72.63%	75.00%	53.33%	35.00%	19.72%
NB	58.33%	54.58%	43.05%	38.19%	18.88%
**Unbalanced Dataset**					

	**Fused**	**Acc**	**Mag**	**Gyro**	**Pressure**

C4.5	94.60%	93.78%	89.25%	77.83%	45.96%
CART	94.73%	94.10%	89.27%	79.27%	47.38%
MLP	94.43%	93.94%	87.53%	78.95%	47.38%
SVM	93.52%	94.50%	89.35%	78.89%	47.38%
NB	64.63%	85.72%	43.62%	68.07%	32.53%

**Table 5.** Smartwatch-based activity results.

**Classifier**	**Result**	

	**Balanced Dataset**	**Unbalanced Dataset**
C4.5	56.89%	88.62%
CART	54.40%	89.26%
MLP	47.89%	87.37%
SVM	55.17%	NA%
NB	51.91%	71.23%

**Table 6.** Overall PCA results for smartphone.

**Classifier**	**Fused**
C4.5	87.55%
CART	89.44%
MLP	92.89%
SVM	92.86%
NB	87.23%

**Table 7.** Overall PCA results for smartwatch.

**Classifier**	**Accelerometer**
C4.5	56.89%
CART	54.40%
MLP	47.89%
SVM	55.17%
NB	51.91%

**Table 8.** Differentiating outdoors from indoors.

	**Fused**	**GPS**	**Light**
C4.5	93.18%	90.90%	88.64%
CART	93.18%	88.63%	84.09%
MLP	95.54%	95.45%	86.36%
SVM	NA	NA	81.81%
NB	100.00%	93.18%	86.36%
